# LncRNA FEZF1-AS1 Promotes TGF-*β*2-Mediated Proliferation and Migration in Human Lens Epithelial Cells SRA01/04

**DOI:** 10.1155/2019/4736203

**Published:** 2019-06-12

**Authors:** Yong Wang, Lili Chen, Yonghui Gu, Ying Wang, You Yuan, Qiujian Zhu, Mingchao Bi, Shuyan Gu

**Affiliations:** ^1^Aier School of Ophthalmology, Central South University, Changsha, Hunan, China; ^2^Department of Ophthalmology, Lixiang Eye Hospital of Soochow University, Suzhou, Jiangsu, China; ^3^Department of Ophthalmology, Suzhou Municipal Hospital, The Affiliated Suzhou Hospital of Nanjing Medical University, Suzhou, Jiangsu, China; ^4^Department of Ophthalmology, The First Hospital, Jilin University, Changchun, Jilin, China

## Abstract

Posterior capsule opacification (PCO) is a common complication after cataract surgery attributed to the proliferation and migration of postoperative residual lens epithelial cells (LECs). The long noncoding RNA (lncRNA) FEZ family zinc finger 1 antisense RNA 1 (FEZF1-AS1) promotes the proliferation and migration of multiple types of cancer cells. Here, we discovered that FEZF1-AS1 is markedly upregulated in TGF-*β*2-treated SRA01/04 cells. In addition, the proliferation and migration of SRA01/04 cells were enhanced following TGF-*β*2 treatment. FEZF1-AS1 knockdown inhibited the TGF-*β*2-induced proliferation and migration of SRA01/04 cells. Accordingly, FEZF1-AS1 overexpression promoted the TGF-*β*2-induced proliferation and migration of SRA01/04 cells. Finally, FEZF1-AS1 upregulated TGF-*β*2-induced SRA01/04 cell proliferation and migration via boosting FEZF1 protein levels. Our findings indicate that the dysregulation of FEZF1-AS1 participates in the TGF-*β*2-induced proliferation and migration of human lens epithelial cells (HLECs), which might be achieved, at least in part, through the induction of FEZF1 expression.

## 1. Introduction

Cataracts are the most common cause of blindness, and cataract surgery is their only cure. Phacoemulsification and extracapsular cataract extraction (ECCE) are the most common types of surgery to treat cataracts. However, posterior capsule opacification (PCO) is a common complication after cataract surgery. Decreased visual acuity induced by PCO occurs in 20–40% of patients 2–5 years postoperatively [1]. PCO is generally associated with the pathological progression of postoperative residual lens epithelial cells (LECs), including proliferation, migration, and epithelial-mesenchymal transition (EMT) [2].

Long noncoding RNAs (lncRNAs), which consist of more than 200 nucleotides (nt) and exhibited limited or no protein-coding capacity [3], are dysregulated in multiple human diseases, including PCO [4, 5]. The long noncoding RNA (lncRNA) FEZ family zinc finger 1 antisense RNA 1 (FEZF1-AS1), which is located on the opposite strand of the FEZF1 gene, upregulates the mRNA level and protein expression of FEZF1 [6]. In addition, FEZF1-AS1 promotes the proliferation and migration of colorectal carcinoma [6], pancreatic ductal adenocarcinoma [7], and osteosarcoma [8]. However, the expression and role of FEZF1-AS1 in the pathogenesis of PCO are unclear.

In this study, we questioned whether FEZF1-AS1 regulated the proliferation and migration of HLECs via modulating FEZF1 expression. Therefore, transforming growth factor-*β*2 (TGF-*β*2)-stimulated HLECs were used to mimic the PCO microenvironment. Quantitative real-time polymerase chain reaction (qRT-PCR) was applied to detect FEZF1-AS1 expression in HLECs following TGF-*β*2 stimulation. Meanwhile, the proliferation and migration of HLECs were detected following TGF-*β*2 stimulation with FEZF1-AS1 knockdown or overexpression. Finally, FEZF1 expression was measured by western blot and inhibited by FEZF1 siRNAs to identify the effect of FEZF1 on the role of FEZF1-AS1. We found that FEZF1-AS1 upregulated TGF-*β*2-induced SRA01/04 cell proliferation and migration via boosting FEZF1 protein levels. Our study demonstrated the novel role and mechanism of FEZF1-AS1 in the pathogenesis of PCO.

## 2. Materials and Methods

### 2.1. Cell Culture and Treatment

SRA01/04 cells that were transformed via SV40 T-antigen [9] were obtained from Jennio Biotech Company (Guangzhou, China). Cells were cultured in Dulbecco's modified Eagle's medium (DMEM) (Gibco/Brl, Grand Island, NY, USA) supplemented with 10% fetal bovine serum (FBS) and incubated at 37°C in a humidified atmosphere containing 5% CO_2_. Cells treated with TGF-*β*2 (Sigma Aldrich, St Louis, USA) at a concentration of 10 ng/ml for 48 h were used as the TGF-*β*2 group.

### 2.2. RNA Isolation and Quantitative Real-Time PCR (qRT-PCR)

Total RNA was extracted using the TRIzol reagent (Invitrogen, Carlsbad, CA, USA). cDNA was synthesized using a PrimeScript RT reagent kit (Promega, Madison, WI, USA). qRT-PCR was performed with SYBR Premix EX Taq™ (Takara, Dalian, China) using an ABI 7500 real-time PCR system (Applied Biosystems, Foster City, USA). GAPDH was used as an endogenous control. Fold changes were calculated through relative quantification (2−∆∆*C*t). The primers used were as follows: for FEZF1-AS1: 5′-TTAGGAGGCTTGTTCTGTGT-3′ and 5′-GCGCAGGTACTTAAGAAAGA-3′ (align to nucleotides 901–920 and 1120–1139 of the FEZF1-AS1 reference sequence NR_036484, respectively), GAPDH: 5′-ACAGTCAGCCGCATCTTCT-3′ and 5′-GACAAGCTTCCCGTTCTCAG-3′ (align to nucleotides 134–152 and 465–485 of the GAPDH reference sequence NM_001289745, respectively), and FEZF1: 5′-CAGGCACAAGATCATTCACACGCAGG-3′ and 5′-CCCTTTTTGATGAAACCCTTTGCCACAG-3′ (align to nucleotides 986–1011 and 1118–1145 of the FEZF1 reference sequence NM_001024613, respectively).

### 2.3. Cell Counting Kit-8 (CCK-8) Assay

SRA01/04 cells (1 × 10^4^ cells/well) were seeded in 96-well plates for 24 h. A CCK-8 kit (Dojindo Laboratories, Japan) was used to analyze cell survival. In brief, 10 *μ*l of CCK-8 solution was added to the cells and incubated for 2 h at 37°C. The absorbance at 450 nm was measured by a microplate reader (BD, USA).

### 2.4. 5-Ethynyl-20-deoxyuridine (EdU) Assay

An EdU kit (Cell Light EdU DNA imaging kit, RiboBio, Guangzhou, China) was used to evaluate cell proliferation according to the manufacturer's instructions. Images were detected and analyzed with a microscope (Olympus, Tokyo, Japan). The average ratio of EdU-stained cells (red) to DAPI-stained cells (blue) was used to evaluate cell proliferation activity.

### 2.5. Western Blot Analysis

Cells were collected and lysed using RIPA protein extraction reagent (Beyotime, Shanghai, China) supplemented with a protease inhibitor cocktail (Roche, Pleasanton, CA, USA) and phenylmethylsulfonyl fluoride (Roche). The concentrations of the protein samples were detected using a BCA protein assay kit (Beyotime). Equal amounts of protein extracts were separated by 10% SDS-PAGE and then transferred to PVDF membranes. The membranes were blocked for 1 h in Tris-buffered saline (TBS) containing 5% nonfat milk and incubated with primary antibodies at 4°C overnight. Autoradiograms were quantified by densitometry using Quantity One software (Bio-Rad, Hercules, CA, USA) with *β*-actin (#60008-1-Ig) as loading control and antibodies against CDK2 (#60312-1-Ig), CDK4 (#11026-1-AP), CDK6 (#19117-1-AP), cyclin D1 (#60186-1-Ig), and FEZF1 (sc-515487, Santa Cruz Biotechnology, Santa Cruz, CA, USA). Except for the FEZF1 antibody, all other antibodies were purchased from Proteintech Group (Chicago, IL, USA).

### 2.6. Wound Healing Assay

**Figure 1:**
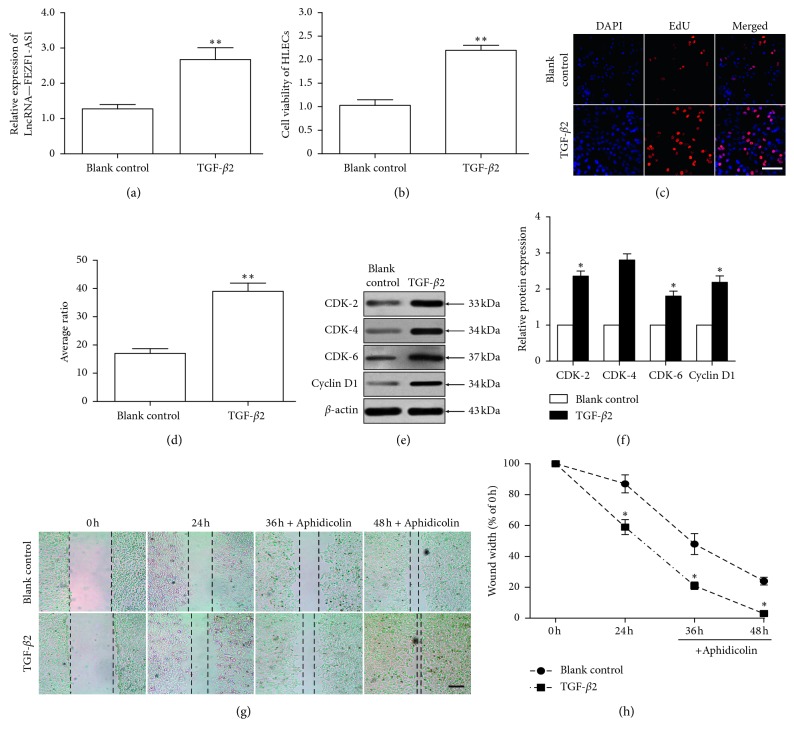
FEZF1-AS1 is upregulated, accompanied by the increased proliferation and migration of TGF-*β*2-treated SRA01/04 cells. The SRA01/04 cells were untreated or treated with 10 ng/ml TGF-*β*2 for 48 h. (a) The FEZF1-AS1 level in SRA01/04 cells was detected by qRT-PCR. (b) The viability of human lens epithelial cells (HLECs, SRA01/04 cells) was detected by CCK-8 assay. (c) SRA01/04 cells were stained with EdU (red) and DAPI (blue). Scale bar = 50 *μ*m. (c) The bar chart shows the average ratio of EdU-stained cells to DAPI-stained cells. CDK2, CDK4, CDK6, and cyclin D1 protein levels were detected by western blot (e) and band density analysis (f). (g) Untreated SRA01/04 cells or SRA01/04 cells treated with TGF-*β*2 for 0 h, 24 h, 36 h, or 48 h were subjected to a wound healing assay to detect migration. Scale bar = 50 *μ*m. (h) The average relative cell migration ratio compared to 0 h was calculated. *n* = 5 in each group. ^*∗∗*^*P* < 0.01, ^*∗*^*P* < 0.05 vs blank control group.

**Figure 2:**
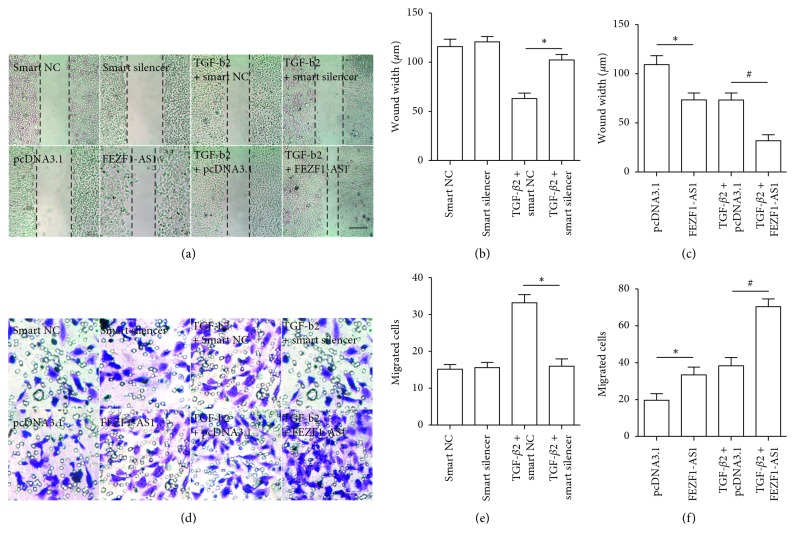
FEZF1-AS1 promotes TGF-*β*2-induced SRA01/04 cell migration. SRA01/04 cells were transfected with smart NC, smart silencer, pcDNA3.1, or pcDNA3.1-FEZF1-AS1 with or without TGF-*β*2 treatment for 48 h. Then, wound healing photos were taken (a), and the wound widths were measured in the FEZF1-AS1 knockdown groups (b) ^*∗*^*P* < 0.05, TGF-*β*2 plus smart silencer group compared to TGF-*β*2 plus smart NC group. (c) The wound widths were measured in the FEZF1-AS1 overexpression groups. ^*∗*^*P* < 0.05, pcDNA3.1-FEZF1-AS1 group compared to pcDNA3.1 group. ^#^*P* < 0.05, TGF-*β*2 plus pcDNA3.1-FEZF1-AS1 group compared to TGF-*β*2 plus pcDNA3.1 group. (d) Representative photos of the transwell assay are shown. (e) The average number of migrated cells was calculated in the FEZF1-AS1 knockdown groups. ^*∗*^*P* < 0.05, TGF-*β*2 plus smart silencer group compared to TGF-*β*2 plus smart NC group. *n* = 5 in each group. (f) The average number of migrated cells was calculated in the FEZF1-AS1 overexpression groups. ^*∗*^*P* < 0.05, pcDNA3.1-FEZF1-AS1 group compared to pcDNA3.1 group. ^#^*P* < 0.05, TGF-*β*2 plus pcDNA3.1-FEZF1-AS1 group compared to TGF-*β*2 plus pcDNA3.1 group. Scale bar = 50 *μ*m in (a) and (c).

SRA01/04 cells were seeded (2 × 10^6^/well) in a 6-well plate. A wound was made by scratching a confluent monolayer with a 200 *μ*l pipette tip. Nonadherent cells were washed off with sterile PBS. The cells were placed in an incubator for 48 h. Pictures were taken by an inverted microscope (Nikon Ti, Japan) at a 50x magnification. The widths of the scratches were measured using Motic Image Plus 2.2S software (Shimadzu, Japan). The relative cell migration compared to the location at 0 h was calculated as a ratio (Figure 1(h)). The wound width (*μ*m) was measured from at least three experiments (Figure 2(b)).

### 2.7. Transfection of SRA01/04 Cells

SRA01/04 cells were seeded into 6-well plates and transfected with smart negative control (NC); smart silencer; scrambled siRNA; FEZF1 siRNA1, FEZF1 siRNA2, or FEZF1 siRNA3 (RiboBio); pcDNA3.1 (empty vector) or pcDNA3.1-FEZF1-AS1 (GenePharma, Suzhou, Jiangsu, China) using Lipofectamine 2000 (Invitrogen, Carlsbad, CA, USA) according to the manufacturer's instructions. The transfected cells were harvested after 48 h for qRT-PCR and western blot analyses.

### 2.8. Transwell Migration Assay

Cell migration was determined by the Transwell assay. After the required treatment, 5 × 10^4^ cells were transferred to the upper chambers of 8 *μ*m hanging inserts in 24-well plates (Corning, USA) in serum-free DMEM. A volume of 800 *μ*l of DMEM containing 10% FBS was then added to the lower chamber. After a 24 h incubation, the noninvaded cells were removed with cotton swabs. The invaded cells were fixed with 4% paraformaldehyde for 15 min and stained with 0.1% crystal violet (Beyotime) for 30 min and photographed. Three different microscopic fields were used to calculate the average number of migrated cells.

### 2.9. Statistical Analysis

All statistical analyses were performed using SPSS 19.0 software (SPSS, USA). Data are represented as the mean ± SEM; all experiments were performed at least in triplicate. Student's *t*-test or one-way analysis of variance (ANOVA) was used for statistical analysis. When a significant difference was apparent by ANOVA, the Dunnett test was used to compare multiple means. A value of *P* < 0.05 indicated statistical significance.

## 3. Results

### 3.1. FEZF1-AS1 Is Upregulated and Accompanied by Increased Proliferation and Migration in TGF-*β*2-Treated SRA01/04 Cells

Using real-time RT-PCR, we first detected FEZF1-AS1 levels and found that FEZF1-AS1 was upregulated in TGF-*β*2-treated SRA01/04 cells compared to its expression in the blank control group (Figure 1(a)). Meanwhile, the viability and proliferation of TGF-*β*2-treated SRA01/04 cells, which were detected by CCK-8 and EdU assays, respectively, increased compared to that observed in control cells (Figures 1(b)–1(d)). Previous studies found that FEZF1-AS1 knockdown repressed the proliferation of gastric cancer and lung adenocarcinoma cells, inhibited cell cycle progression by causing G1/S arrest, and decreased the levels of cyclin-dependent kinase 2 (CDK2), cyclin-dependent kinase 4 (CDK4), cyclin-dependent kinase 6 (CDK6), and cyclin D1 [10, 11]. Interestingly, western blotting demonstrated that CDK2, CDK4, CDK6, and cyclin D1 protein levels were upregulated in the TGF-*β*2-treated cells compared to their expression in the blank control cells (Figures 1(e) and 1(f)). Additionally, the wound healing assay showed that cell migration was increased in the TGF-*β*2-treated cells compared to that in the control cells (Figures 1(g) and 1(h)). These data suggested that FEZF1-AS1 was upregulated and accompanied by the increased proliferation and migration of TGF-*β*2-treated SRA01/04 cells, implying that FEZF1-AS1 was associated with the proliferation and migration of SRA01/04 cells after TGF-*β*2 stimulation.

### 3.2. FEZF1-AS1 Promotes TGF-*β*2-Induced SRA01/04 Cell Proliferationv

**Figure 3:**
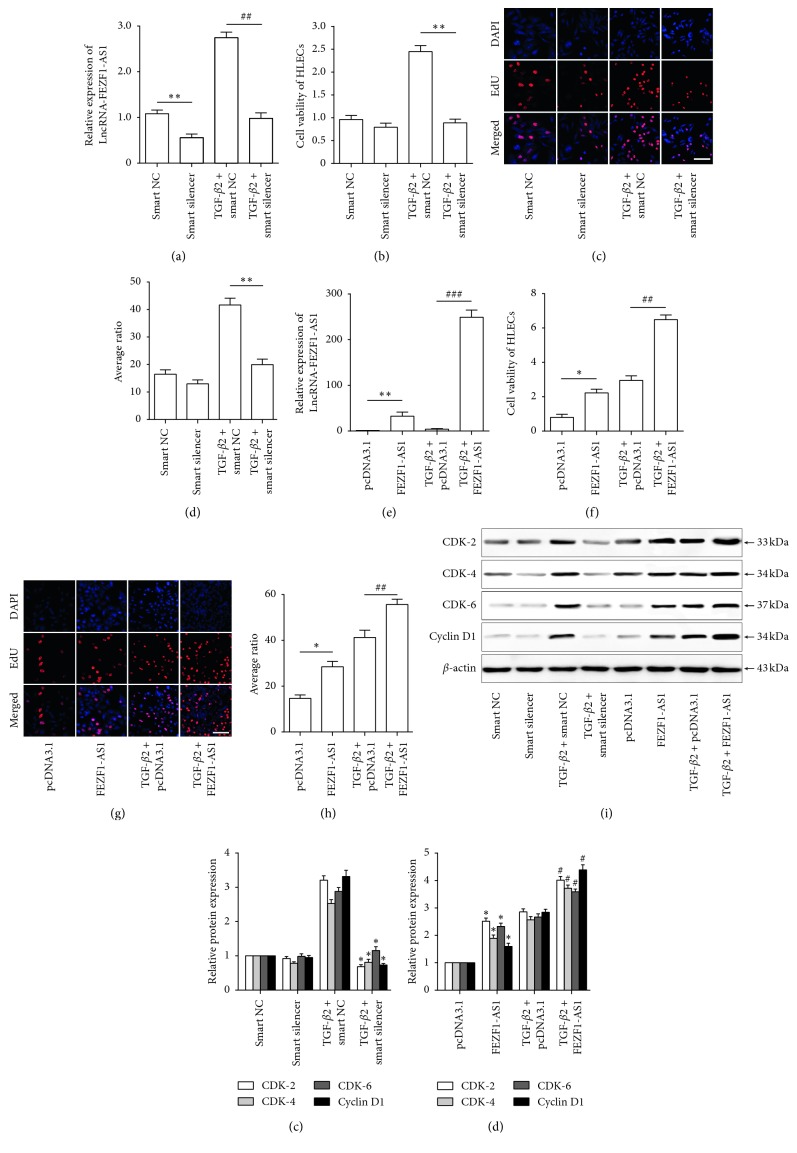
FEZF1-AS1 promotes TGF-*β*2-induced SRA01/04 cell proliferation. (a) SRA01/04 cells were transfected with smart negative control (NC) or smart silencer with or without TGF-*β*2 treatment for 48 h. FEZF1-AS1 levels in SRA01/04 cells were detected by qRT-PCR. ^*∗∗*^*P* < 0.01, smart silencer group vs smart NC group. ^##^*P* < 0.01, TGF-*β*2 plus smart silencer group vs TGF-*β*2 plus smart NC group. SRA01/04 cells were transfected with smart NC or smart silencer with or without TGF-*β*2 treatment for 48 h. (b) The viability of HLECs (SRA01/04 cells) was detected by CCK-8 assay. ^*∗∗*^*P* < 0.01 vs TGF-*β*2 plus smart NC group. (c) SRA01/04 cells were stained with EdU (red) and DAPI (blue). (d) The bar chart shows the average ratio of EdU-stained cells to DAPI-stained cells. ^*∗∗*^*P* < 0.01, TGF-*β*2 plus smart silencer group vs TGF-*β*2 plus smart NC group. (e) SRA01/04 cells were transfected with pcDNA3.1 (empty vector) or pcDNA3.1-FEZF1-AS1 with or without TGF-*β*2 treatment for 48 h. FEZF1-AS1 levels in SRA01/04 cells were detected by qRT-PCR. ^*∗∗*^*P* < 0.01, pcDNA3.1-FEZF1-AS1 group vs pcDNA3.1 group. ^###^*P* < 0.001, TGF-*β*2 plus pcDNA3.1-FEZF1-AS1 group vs TGF-*β*2 plus pcDNA3.1 group. SRA01/04 cells were transfected with pcDNA3.1 or pcDNA3.1-FEZF1-AS1 with or without TGF-*β*2 treatment for 48 h. (f) The viability of HLECs (SRA01/04 cells) was detected by CCK-8 assay. ^*∗*^*P* < 0.05, pcDNA3.1-FEZF1-AS1 group vs pcDNA3.1 group. ^##^*P* < 0.01, TGF-*β*2 plus pcDNA3.1-FEZF1-AS1 group vs TGF-*β*2 plus pcDNA3.1 group. (g) SRA01/04 cells were stained with EdU (red) and DAPI (blue). (h) The bar chart shows the average ratio of EdU-stained cells to DAPI-stained cells. ^*∗*^*P* < 0.05 vs pcDNA3.1 group. ^##^*P* < 0.01 vs TGF-*β*2 plus pcDNA3.1 group. SRA01/04 cells were transfected with smart NC, smart silencer, pcDNA3.1, or pcDNA3.1-FEZF1-AS1 with or without TGF-*β*2 treatment for 48 h. CDK-2, CDK-4, CDK-6, and cyclin D1 protein levels were detected by western blot and (i) band density analysis (j) ^*∗*^*P* < 0.05, TGF-*β*2 plus smart silencer group compared to TGF-*β*2 plus smart NC group. (k) ^*∗*^*P* < 0.05, pcDNA3.1-FEZF1-AS1 group compared to pcDNA3.1 group. ^#^*P* < 0.05, TGF-*β*2 plus pcDNA3.1-FEZF1-AS1 group compared to TGF-*β*2 plus pcDNA3.1 group. *n* = 5 in each group. Scale bar = 50 *μ*m in (c) and (g).

To identify the role of FEZF1-AS1 in TGF-*β*2-induced SRA01/04 cell proliferation, a FEZF1-AS1 smart silencer was transfected into normal and TGF-*β*2-treated SRA01/04 cells to downregulate the FEZF1-AS1 level (Figure 3(a)). FEZF1-AS1 knockdown inhibited the TGF-*β*2-induced increase in the viability and proliferation of SRA01/04 cells but did not affect untreated cells (Figures 3(b)–3(d)). Furthermore, we also used pcDNA3.1-FEZF1-AS1 to overexpress FEZF1-AS1 in normal and TGF-*β*2-treated SRA01/04 cells (Figure 3(e)). FEZF1-AS1 overexpression increased the viability and proliferation of SRA01/04 cells independent of TGF-*β*2 treatment (Figures 3(f)–3(h)). The TGF-*β*2-induced CDK2, CDK4, CDK6, and cyclin D1 protein levels were downregulated by FEZF1-AS1 knockdown and upregulated by FEZF1-AS1 overexpression, but only FEZF1-AS1 overexpression increased CDK2, CDK4, CDK6, and cyclin D1 protein levels in untreated cells (Figures 3(i)–3(k)). These results suggested that FEZF1-AS1 promoted TGF-*β*2-induced SRA01/04 cell proliferation at least partly via modulating the cell cycle.

### 3.3. FEZF1-AS1 Promotes TGF-*β*2-Induced SRA01/04 Cell Migration

We next used wound healing (Figures 2(a)–2(c)) and Transwell assays (Figures 2(c)–2(e)) following FEZF1-AS1 knockdown or overexpression to explore the role of FEZF1-AS1 in TGF-*β*2-induced SRA01/04 cell migration, which showed that FEZF1-AS1 promoted TGF-*β*2-induced SRA01/04 cell migration.

### 3.4. FEZF1-AS1 Promotes TGF-*β*2-Induced SRA01/04 Cell Proliferation and Migration via Upregulating FEZF1 mRNA and Protein Levels

**Figure 4:**
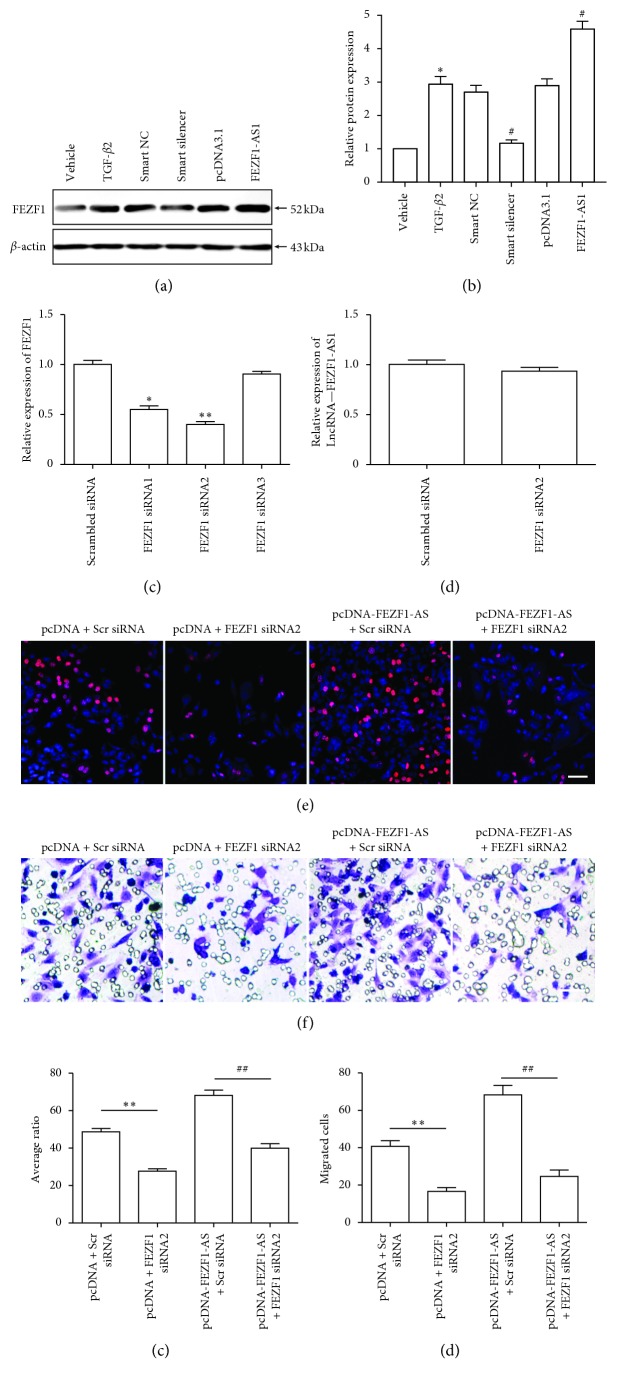
FEZF1-AS1 promotes TGF-*β*2-induced SRA01/04 cell proliferation and migration via upregulating FEZF1 protein levels. (a) The FEZF1 protein level in the vehicle, TGF-*β*2-treated, TGF-*β*2 plus smart NC, TGF-*β*2 plus smart silencer, TGF-*β*2 plus pcDNA3.1, and TGF-*β*2 plus pcDNA3.1-FEZF1-AS1 SRA01/04 cells was detected by western blot. (b) The intensity of the band for FEZF1 was analyzed. ^*∗*^*P* < 0.05, TGF-*β*2-treated group vs vehicle group. ^#^*P* < 0.05, TGF-*β*2 plus smart silencer group or TGF-*β*2 plus pcDNA3.1-FEZF1-AS1 group vs TGF-*β*2 group. (c) The FEZF1 mRNA levels in SRA01/04 cells transfected with scrambled siRNA, FEZF1 siRNA1, FEZF1 siRNA2, or FEZF1 siRNA3 were detected by qRT-PCR. ^*∗*^*P* < 0.05, ^*∗∗*^*P* < 0.01, vs scrambled siRNA group. (d) The FEZF1-AS1 levels in SRA01/04 cells transfected with scrambled siRNA or FEZF1 siRNA2 were detected by qRT-PCR. (e) SRA01/04 cells transfected with pcDNA3.1 plus scrambled siRNA, pcDNA3.1 plus FEZF1 siRNA2, pcDNA3.1-FEZF1-AS1 plus scrambled siRNA, or pcDNA3.1-FEZF1-AS1 plus FEZF1 siRNA2 were subjected to EdU ((e) and (g)) and transwell assays ((f) and (h)). ^*∗∗*^*P* < 0.01, pcDNA3.1 plus FEZF1 siRNA2 group vs pcDNA3.1 plus scrambled siRNA in (g) and (h). ^##^*P* < 0.01, pcDNA3.1-FEZF1-AS1 plus FEZF1 siRNA2 group vs pcDNA3.1-FEZF1-AS1 plus scrambled siRNA group in (g) and (h). *n* = 5 in each group. Scale bar = 50 *μ*m in (e) and (f).

Previous studies found that FEZF1-AS1 increased FEZF1 protein levels [6, 12]. Thus, we speculated that FEZF1-AS1 plays a positive role in TGF-*β*2-induced SRA01/04 cell proliferation and migration via upregulating FEZF1 protein levels. FEZF1-AS1 knockdown inhibited (Figure 4(a)) FEZF1 protein levels, and its overexpression increased FEZF1 protein levels (Figures 4(a) and 4(b)). Moreover, FEZF1 siRNAs were also used to downregulate the FEZF1 mRNA level (Figure 2(c), FEZF1 siRNA2 showed the best efficiency and was used in subsequent experiments) without affecting the FEZF1-AS1 level (Figure 4(d)). In SRA01/04 cells, FEZF1 siRNA2 inhibited TGF-*β*2- and FEZF1-AS1-induced proliferation (Figures 4(e) and 4(g)) and migration (Figures 4(f) and 4(h)). These results suggested that FEZF1-AS1 promoted the proliferation and migration of TGF-*β*2-induced SRA01/04 cells via upregulating FEZF1 protein levels.

## 4. Discussion

PCO is a common and significant complication following cataract surgery. At present, surgical intervention is the only cure for cataracts [1]; however, after cataract surgery, the growth of aberrant lens epithelial cells (LECs) across the lens capsule often leads to migration, fibrosis, and collagen deposition, leading to secondary visual loss known as PCO. Cell culture is the simplest method to study PCO and generally utilizes cell lines to analyze PCO characteristics. Experiments using these model systems permit the determination of factors that stimulate or inhibit proliferation, migration, differentiation, transdifferentiation, and matrix contraction [13]. SRA01/04 cells express mRNA for multiple growth factor receptors including TGF-*β*2, epidermal growth factor (EGF), and insulin-like growth factor 1 (IGF-1) [13]. SRA01/04 cells also express integrins *α*2 and *α*3. Integrin *α*2*β*1 is a receptor for laminin, collagen, fibronectin, and E-cadherins. However, integrin *α*3*β*1 binds to matrix proteins such as collagen type IV and laminin, which are major components of the lens capsule [14]. These data provide a basis for the use of the SRA01/04 cell line as a model to study PCO in vitro. Transforming growth factor *β* (TGF*β*) and especially TGF-*β*2, the major isoform in the aqueous humor of the eye, play a central role in the pathogenesis of PCO [15, 16]. In our study, treatment with TGF-*β*2 at a concentration of 10 ng/ml for 48 h was used to stimulate SRA01/04 cells [17, 18], mimicking PCO in vitro.

In our study, TGF-*β*2 induced the expression of FEZF1-AS1 in SRA01/04 cells (Figure 1(a)). Currently, there is no publication about the upstream transcription factors or modulators that regulate FEZF1-AS1 expression. Activin/TGF-*β*-specific, receptor-regulated Smad 3 (Smad3) is an intracellular signal transducer and transcriptional modulator activated by TGF-*β*2 and activin type 1 receptor kinases [19]. TGF-*β*2 induces the phosphorylation of Smad3. Following its phosphorylation, Smad3 translocates to the nucleus and promotes the transcription of lncRNAs [20]. For example, the lncRNA LINC01186, which inhibits migration and invasion via epithelial-mesenchymal transition (EMT) in lung cancer, is regulated by TGF-*β*/Smad3 [21]. Another example is ErbB4-immunoreactivity (Erbb4-IR), which is a novel lncRNA that contributes to TGF-*β*/Smad3-mediated renal fibrosis and a potential therapeutic target for chronic fibrotic kidney disease [22]. Therefore, we speculated that the TGF-*β*2/Smad3 signaling pathway regulates FEZF1-AS1 expression.

Interestingly, we found that TGF-*β*2 induced SRA01/04 cell proliferation (Figures 1(b)–1(d)), which was consistent with previous studies [23, 24]. However, some studies discovered that TGF-*β*2 suppressed the proliferation of HLE-B3 cells, which are another HLEC line, and primary HLECs [25]. This contradiction can be attributed to several things. First, the dose and stimulation time used were different. TGF-*β*2 at a concentration of 10 ng/ml was used for 48 h in our study, and 1 ng/ml TGF-*β*2 was used for 12 h in their study. Second, the cell lines were different. SRA01/04 cells were utilized in our study, while HLE-B3 cells and primary HLECs were utilized in their study. Four microRNAs (miRNAs), including miR-31, miR-124, miR-184, and miR-222, are differentially expressed between SRA01/04 and HLE-B3 cells [26]. These differences in miRNA expression might promote different TGF-*β*2-induced and proliferation-associated patterns of gene expression. As a result, TGF-*β*2 induces a distinct proliferation response in the two HLEC cell lines. Finally, the cell proliferation assays used were different. We used CCK-8 and EdU incorporation assays, and the authors of the other studies performed a colorimetric WST-1 assay and proliferating cell nuclear antigen (PCNA) western blot. Which signaling pathways downstream of TGF-*β*2 in different HLECs are related to cell proliferation? The question requires future study.

The Fez family zinc finger protein 1 (FEZF1) is a C_2_H_2_ zinc finger transcription factor that plays critical roles in the development of the forebrain and olfactory system in vertebrates [27]. In mice, FEZF1 binds to and represses the expression of Hes family BHLH transcription factor 5 (Hes5), a transcription factor that inhibits neuronal differentiation and facilitates neurogenesis in the forebrain [28]. FEZF1 has also been implicated in the progression of human cancers. In gastric cancer cells, FEZF1 enhances proliferation and tumorigenic by binding to and activating Kirsten rat sarcoma viral oncogene homolog (KRAS) [29]. In addition, FEZF1 promotes cell migration and invasion in colorectal cancer cells [6]. We also found that FEZF1-AS1 promotes TGF-*β*2-induced SRA01/04 cell proliferation and migration via upregulating FEZF1 protein levels. In an in vitro experiment, FEZF1 promoted the proliferation, migration, and invasion of glioma cells and inhibited cell apoptosis by activating the protein kinase B alpha (Akt)-extracellular signal-regulated kinase (ERK) pathway [30]. The growth factor (such as EGF) induced proliferation of HLECs in the aqueous humor is dependent on the mitogen-activated protein kinase (MAPK)/ERK and Akt/PI3K signaling pathways [31]. Furthermore, EGF-induced cell migration is mediated by the ERK and phosphatidylinositol-3 kinase (PI3K)/AKT pathways in cultured HLECs [32]. Thus, we speculate that FEZF1 promotes the proliferation and migration of HLECs via upregulating the ERK and AKT signaling pathways, which requires intensive investigation.

We also found that FEZF1-AS1 upregulated FEZF1 protein levels (Figures 4(a) and 4(b)). FEZF1-AS1 increased the aggressive behavior of colorectal carcinoma cells by increasing the mRNA levels of its corresponding cognate gene, FEZF1, via modulating the transcription of FEZF1 or the stability of its mRNA [6], suggesting the mechanism by which FEZF1-AS1 upregulated FEZF1 protein levels. The detailed mechanism underlying the upregulation of FEZF1 expression FEZF1-AS1 requires future exploration.

In addition to upregulating FEZF1 expression, FEZF1-AS1 promoted cell proliferation and migration through other mechanisms. For example, the downregulation of FEZF1-AS1 inhibited the proliferation of gastric cancer (GC) cells, arrested the cell cycle at the G0/G1 stage, and suppressed the activation of the wingless (Wnt)/*β*-catenin signaling pathway [33]. The Wnt/*β*-catenin signaling pathway participates in cell proliferation, differentiation, and migration [34]. Wnt3a, an outstanding member of the Wnt family, induces the accumulation of *β*-catenin and the activation of the Wnt/*β*-catenin signaling pathway [35]. Wnt3a boosts EMT in HLE-B3 cells and their migration and proliferation [36]. Additionally, Dickkopf-1 (Dkk1) inhibits Wnt3a-induced migration and EMT in HLE-B3 cells [37]. Whether FEZF1-AS1 promotes the proliferation and migration of HLECs via the activation of the Wnt/*β*-catenin pathway awaits further investigation.

However, there were several limitations of our study that should be noted; primary HLECs were not used, and FEZF1 mRNA levels were not detected. In summary, our study discovered that FEZF1-AS1 promotes the proliferation and migration of HLECs via upregulating FEZF1 expression, implying a novel therapeutic target for PCO.

## Data Availability

The data used to support the findings of this study are available from the corresponding author upon request.
